# Supplementary material for: Content-rich biological network constructed by mining PubMed abstracts
Source: BMC Bioinformatics. 2004 Oct 8;5:147. doi: 10.1186/1471-2105-5-147 (PMC528731; doi:10.1186/1471-2105-5-147)
Supplement: Additional File 2 — The original results of the above study (non-essential files are deleted to keep the file size under the limit set by BMC bioinformatics). [file 1471-2105-5-147-S2.bz2 › chilibotAdditionalFile2/dip40/472ID9195914E10538/html/IL8RA_CXCL1.html]

 


 **IL8RA** and **CXCL1** 
  
Found 47 abstracts in PubMed, retrieved 40.  
 

 What does Google say? 
 PDF only 
| .edu only 

---

**Interactive relationship** (e.g. stimulation, inhibition, etc)

**Inhibitory relationship**- Substitution into IL 8 of the GRO  [ **CXCL1** ]  gamma sequences corresponding to either the amino terminal loop amino acids 1 18 or the first beta sheet amino acids 18 32 reduced binding to both IL8R1  [ **IL8RA** ]  and IL8R2.  Ref: 8626516 J Biol Chem, 1996
**Neutral relationship**- The chemokines interleukin 8 IL 8 and GRO  [ **CXCL1** ]  alpha bind in neutrophils to the interleukin 8 receptor alpha  [ **IL8RA** ]  and beta IL 8R alpha and beta triggering reorganization of the actin cytoskeleton and activation of phospholipase C PLC.  Ref: 8689391 Inflamm Res, 1996
- CXCR1  [ **IL8RA** ]  and CXCR2 are receptors for ELR positive CXC chemokines which include growth regulated oncogene alpha GRO  [ **CXCL1** ]  alpha and interleukin 8 IL 8.  Ref: 11356265 Brain Res Dev Brain Res, 2001
- Together, these results show that a CXC chemokine such as GRO  [ **CXCL1** ]  alpha can promote malignant growth of murine squamous cell carcinoma by a host CXCR  [ **IL8RA** ]  2 dependent pathway.  Ref: 10918606 Oncogene, 2000

**Non-interactive relationship** (e.g. studied together, co-existance, homology, etc.)

- When IL 8 sequences were substituted into GRO  [ **CXCL1** ]  gamma, a single domain containing the second beta sheet of IL 8 amino acids 18 32 was sufficient to confer high affinity binding for both IL8R1  [ **IL8RA** ]  and IL8R2.  Ref: 8626516 J Biol Chem, 1996
- To determine the regions of interleukin 8 IL 8 that allow high affinity and interleukin 8 receptor type 1  [ **IL8RA** ]  IL8R1  [ **IL8RA** ]  specific binding of chemokines, we produced chimeric proteins containing structural domains from IL 8, which binds to both IL8R1  [ **IL8RA** ]  and interleukin 8 receptor type 2 IL8R2 with high affinity, and from GRO  [ **CXCL1** ]  gamma, which does not bind to IL8R1  [ **IL8RA** ]  and binds to IL8R2 with reduced affinity.  Ref: 8626516 J Biol Chem, 1996
- In addition, we demonstrate that compounds which inhibit the actions of IL 8 and GRO  [ **CXCL1** ]  alpha via their receptors, CXCR1  [ **IL8RA** ]  and CXCR2, also inhibit HIV 1 replication in both T lymphocytes and macrophages, indicating potential therapeutic uses for these compounds in HIV 1 infection and AIDS.  Ref: 11483765 J Virol, 2001
- The response to IL 8 was inhibited slightly by anti IL8R1  [ **IL8RA** ]  and more substantially when both monoclonal antibodies were present, while the response to GRO  [ **CXCL1** ]  alpha was inhibited by anti IL8R2 but was not affected by anti IL8R1  [ **IL8RA** ] .  Ref: 8692878 Proc Natl Acad Sci U S A, 1996
- At 10 nM and higher concentrations, GCP 2 and IL 8 induced significant activation of CXCR1  [ **IL8RA** ]  expressing cells, but no activity was found with GRO  [ **CXCL1** ]  alpha and ENA 78.  Ref: 9485196 Eur J Immunol, 1998
- PMNs were exposed to interleukin 8 IL 8, growth regulated oncogene alpha GRO  [ **CXCL1** ]  alpha, and platelet activating factor PAF to stimulate the CXCR1  [ **IL8RA** ] , CXCR2, and PAF receptors.  Ref: 11130495 J Trauma, 2000
- For example strong calcium responses were seen in neutrophils following stimulation with the CXCR1  [ **IL8RA** ]  and CXCR2 ligands, interleukin IL 8, GCP 2 and Gro  [ **CXCL1** ]  beta.  Ref: 11298490 Cytokine, 2001
- Melanoma growth stimulatory activity  [ **CXCL1** ]  growth related protein alpha MGSA  [ **CXCL1** ]  GROalpha, which binds CXCR2 but not CXCR1  [ **IL8RA** ] , was unable to either stimulate IL 8 secretion in MNCs or desensitize these cells to respond to immobilized IL 8.  Ref: 11076803 Am J Physiol Lung Cell Mol Physiol, 2000
- Using Jurkat cells that stably express either CXCR1  [ **IL8RA** ]  or CXCR2, we studied receptor activation by IL 8, GCP 2 epithelial neutrophil activating protein 2 ENA 78 which shares 77% identical amino acids with GCP 2 and growth regulated oncogene alpha GRO  [ **CXCL1** ]  alpha.  Ref: 9485196 Eur J Immunol, 1998
- Two monoclonal antibodies, anti IL8R1  [ **IL8RA** ]  and anti IL8R2, raised against both interleukin 8 receptors IL 8R of human neutrophils, IL 8R1 and IL 8R2, were used to study individual receptor functions after stimulation with IL 8, GRO  [ **CXCL1** ]  alpha, or NAP 2.  Ref: 8692878 Proc Natl Acad Sci U S A, 1996
- Selective CXCR2 antagonists may be useful to block the Gro  [ **CXCL1** ]  alpha induced priming response whilst preserving neutrophil functions mediated by CXCR1  [ **IL8RA** ] , the alternative neutrophil receptor for interleukin 8.  Ref: 11589350 Eur Respir J, 2001
